# The Second Carey Coombs Memorial Lecture. "The Radiology of Rheumatic Heart Disease"

**Published:** 1949-04

**Authors:** John Parkinson

**Affiliations:** Physician, National Heart Hospital, London; Consulting Physician, Cardiac Department, London Hospital


### THE SECOND
### CAREY COOMBS MEMORIAL LECTURE*

BY

Sir John Parkinson, M.D., F.R.C.P.

*Physician, National Heart Hospital, London;*

*Consulting Physician, Cardiac Department, London Hospital.*

DELIVERED IN THE UNIVERSITY OF BRISTOL
ON JANUARY 27th, 1949

ON

### THE RADIOLOGY OF RHEUMATIC HEART DISEASE

---

The lecturer opened with a tribute to the work of Dr. Carey Coombs on the ætiology of rheumatic heart disease. He pointed out that development of X-ray investigation of the heart had been extended by the introduction of angeiocardiography: that is, injection into a vein of a substance opaque to X-rays followed immediately by taking X-ray films of the heart.

Angeiocardiography has clarified our ideas on the X-ray appearances of the normal heart. The left middle or pulmonary arc used to be regarded as the shadow of the pulmonary artery, but we know now that this shadow is largely that of the left branch. The position of the valves has been studied in patients with calcification of the valves: the aortic valve is seen in approximately the centre of the heart's shadow, the mitral valve is close to it but lower and to the left. The left auricle may be called the posterior auricle, certainly in the right oblique view. The left oblique view is the best to demonstrate the course of the aorta.

Angeiocardiography is of special value in demonstrating the chambers of the heart. Radioscopy is considerably more accurate than percussion. The position of the apex beat, determined by palpation, may be misleading: thus the rapid, forcible beat in hyperthyroidism may lead to a false diagnosis of enlargement. So too in rheumatic carditis, X-rays have shown that enlargement of

*Although this was announced as the "Inaugural" Lecture, the first was delivered in the University on May 5th, 1937, and reported in our pages, Vol. LIV. (No. 204), p. 109–126.

the heart may occur during the disease, but not early as has been thought. The ventricle enlarges backwards as well as outwards, and this is best seen in the left oblique view, which well shows the changes in shape of the cardiac outline.

In radioscopy in the left oblique position, the "angle of clearance" is useful: i.e., the amount of rotation needed to bring the shadow of the left ventricle clear of that of the spine; if this angle exceeds 55°, enlargement is confirmed. A normal contour is said to be inconsistent with a diagnosis of carditis. But enlargement does not necessarily connote active rheumatic disease, for it may be the result of a previous attack. If enlargement has occurred it may remain, increase, or regress.

Calcified valves have been observed at post-mortem for years; they are now easily discerned on the screen under X-rays. Look first in the region of the aortic valves, next in that of the mitral. Aortic valvular disease was found in 202 out of 609 recruits with early rheumatic heart disease. Dr. Carey Coombs had estimated that one-half of all patients with rheumatic heart disease had an aortic valvular lesion. Aortic stenosis is a late result of rheumatic carditis occurring in childhood. Enlargement of the heart may be the first evidence of aortic incompetence.

Mitral incompetence is not now considered of great clinical importance; most mitral valvular disease is mitral stenosis. In mitral stenosis the upper half of the heart shadow is wide owing to the left auricle extending to right and left. Left auricular enlargement is, however, best seen in the right oblique position, with some barium in the œsophagus. Sometimes it is possible to see the outline of the auricle itself through the heart shadow.

Aortic incompetence with mitral stenosis form a combination not often enough diagnosed during life, though often discovered at necropsy.

In conclusion, the lecturer showed the value of angeiocardiography in the diagnosis of aortic aneurysms, some of which would otherwise be quite unsuspected during life. The lecture was illustrated by a large selection of lantern slides demonstrating the varied use of X-rays in cardiology.

